# Supplementary material for: Dissecting the NK Cell Population in Hematological Cancers Confirms the Presence of Tumor Cells and Their Impact on NK Population Function
Source: Vaccines (Basel). 2020 Dec 2;8(4):727. doi: 10.3390/vaccines8040727 (PMC7761578; doi:10.3390/vaccines8040727)

Supplemental Figure 1. Gating strategy for conventional flow cytometry analysis. A) Representative FACS plots showing manual gating for immune subsets in AML patients. B) Representative FACS plots showing manual gating for immune subsets in HL patients.

A

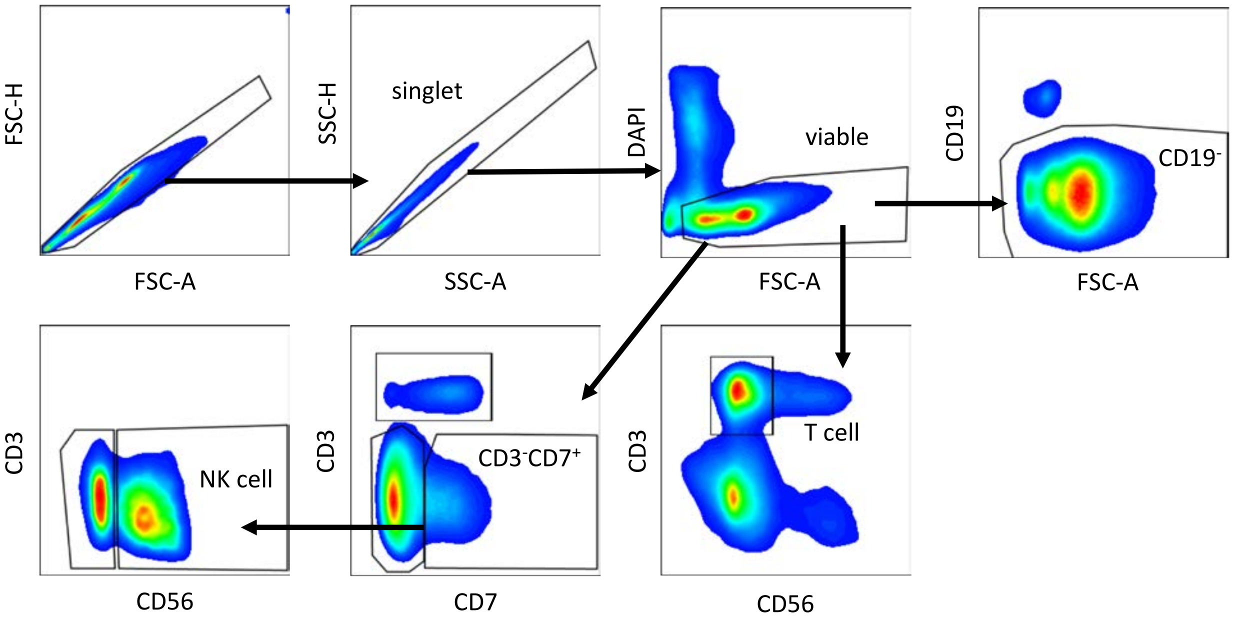

B

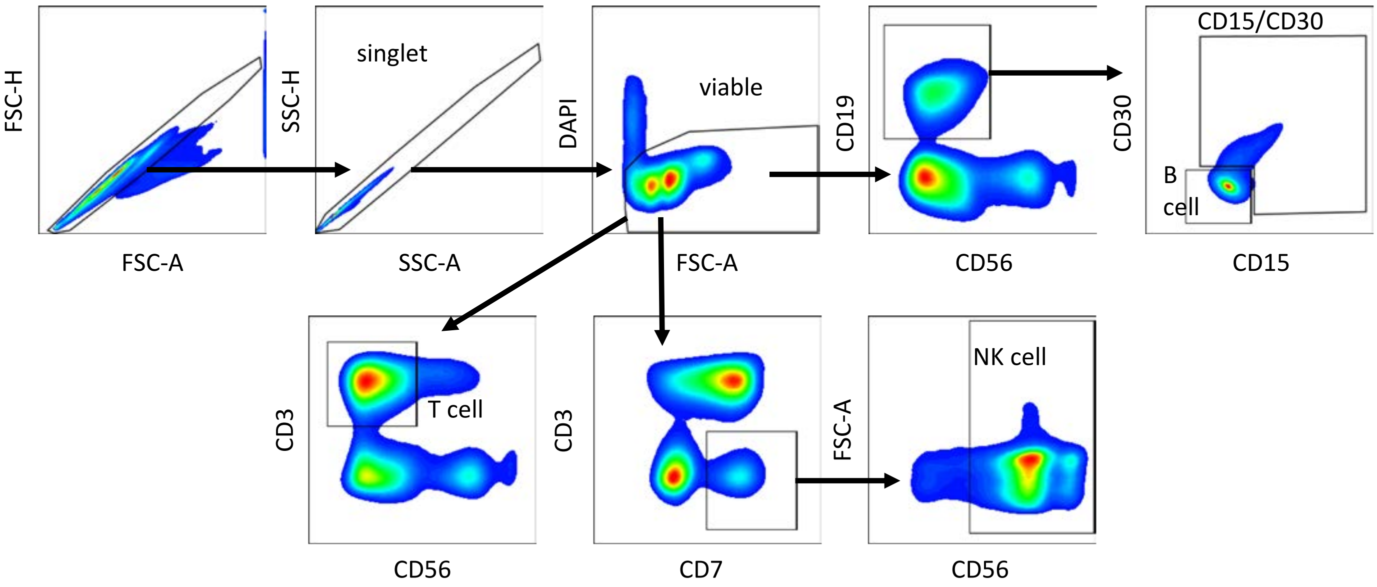

Supplemental Figure 2. Proportions of immune subsets on total HL patients before and after treatment. A) Schema of the study design. Patient's PBMC were collected and analyzed by flow cytometry at diagnosis and after treatment. B) Percentage of lymphocytes. Proportion of B (CD19+), T (CD3+CD56-), NK cell (CD56+ CD7+CD3-) and CD15/CD30+ B cells. C) Total blood count number of immune populations. Significance was determined by paired t-test between diagnosis and at the end of treatment; n = 21, * p ≤ 0.05, ** p ≤ 0.01 and *** p ≤ 0.001.

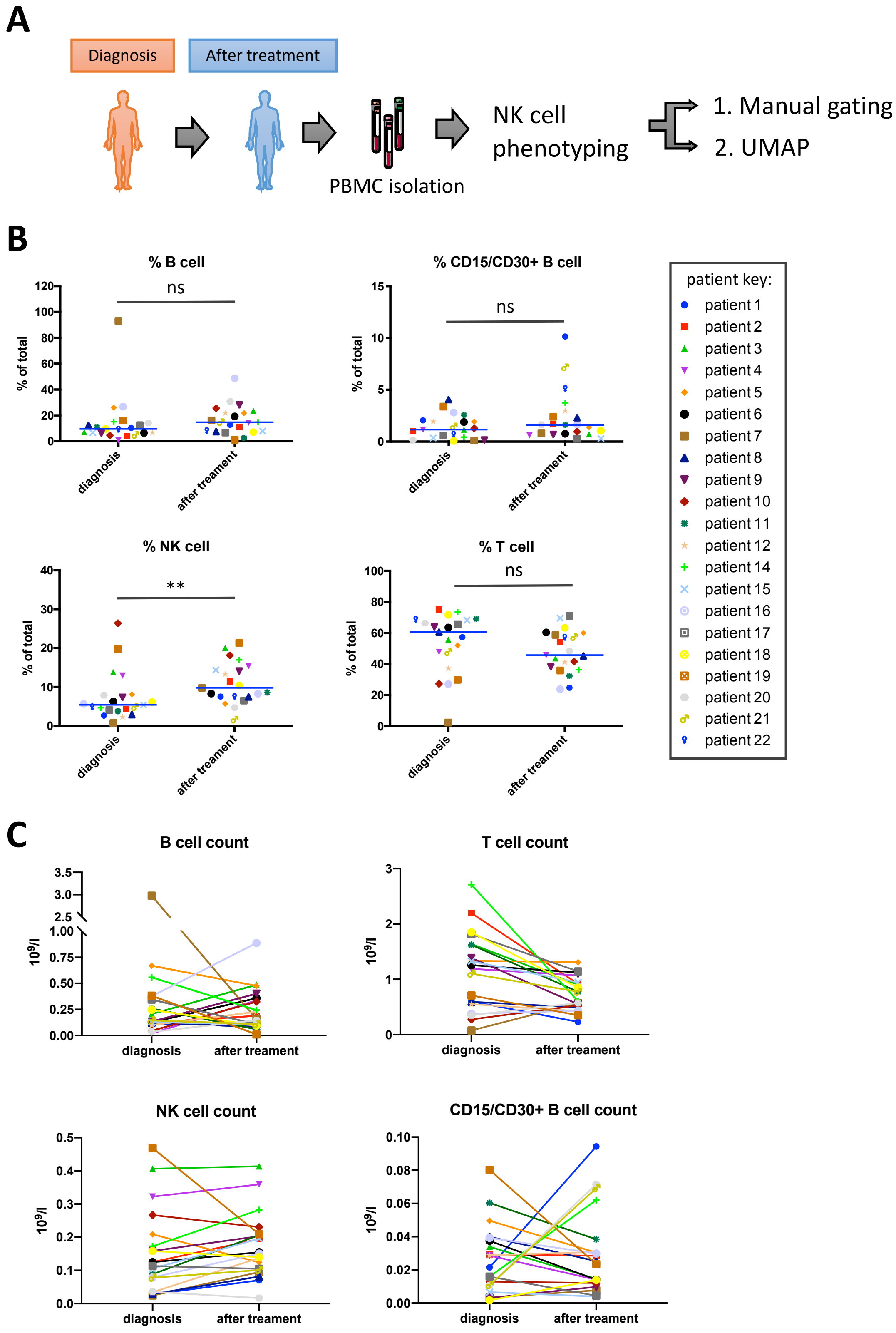

Supplemental Figure 3. CD7 is a marker for conventional NK cells. FACS plot from a representative HD showing CD7 expression on peripheral CD56+ cells. The right bar plot represents the average of CD7+ cells on CD56+ cells in our HD cohort (n=4).

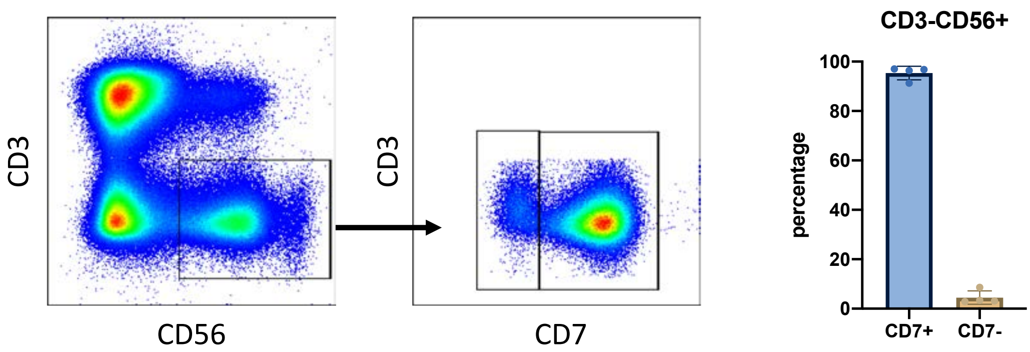

Supplemental Figure 4. Changes in NK cell phenotype is independent of treatment. A) HL patients described in Table 1 were stratified in three different treatment regimes: PVAB (patients 4, 5, 8, 14, 19), ABVD (patients 1, 9, 11, 12, 15, 16, 17, 18), EACOPP/BEACOPP (patients 2, 6, 7, 10, 21 and 22). The percentages of CD45RARO, CD107a+ and CD15+ or CD30+ NK cells were quantified as in Figure 2. B) AML patients described in Table 2 were stratified in three different treatment regimes: Ida+Cyta = Idarubicine + Cytarabine 1 induction (I) (patients 8, 10 ,14); Ida+Cyta 2 inductions (II) patients 2 and 15; Ida+Cyta+Aza = Idarubicine + Cytarabine + Azacitidine, patient 20.

A

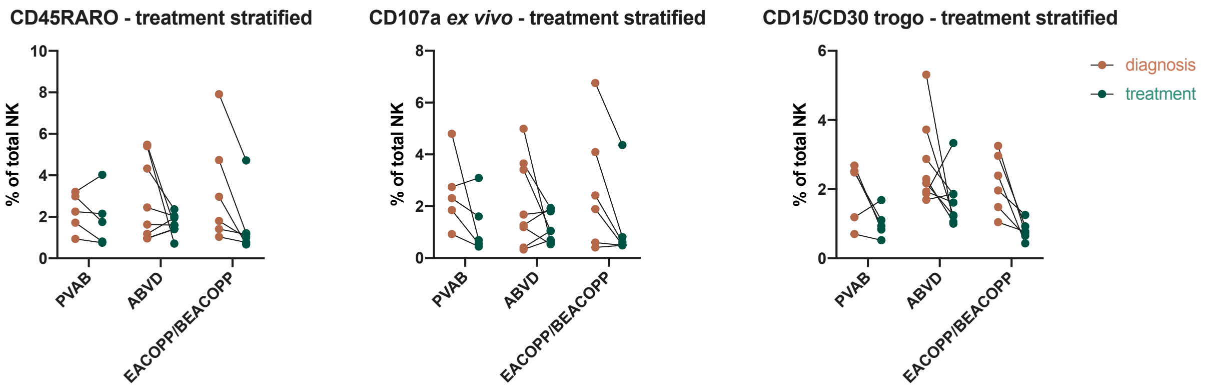

B

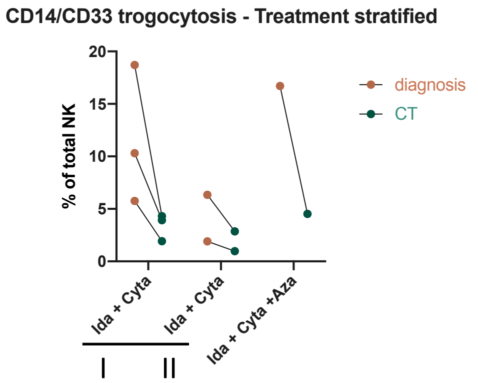

Supplemental Fig. 5. Heatmaps of the expression of the 14 surface markers used to constitute the original UMAP embed.

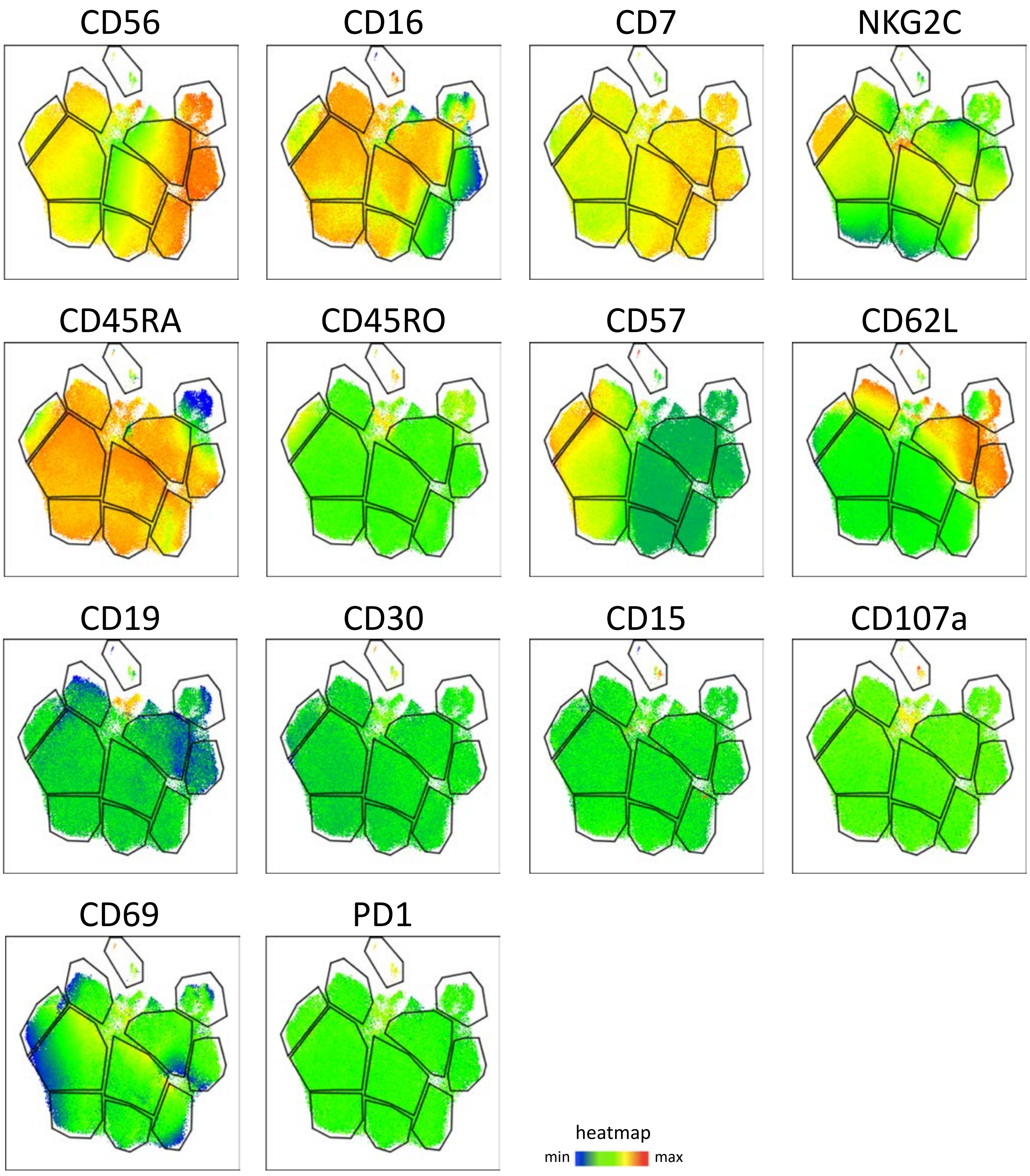

Supplemental Figure 6. Effect of treatment on NK cell clusters in HL patients. Percentage of each cluster before and after treatment for every cluster identified in UMAP. Statistical significance determined by paired t-test, n = 21, * p ≤ 0.05, ** p ≤ 0.01 and *** p ≤ 0.001, unless exact value otherwise indicated.

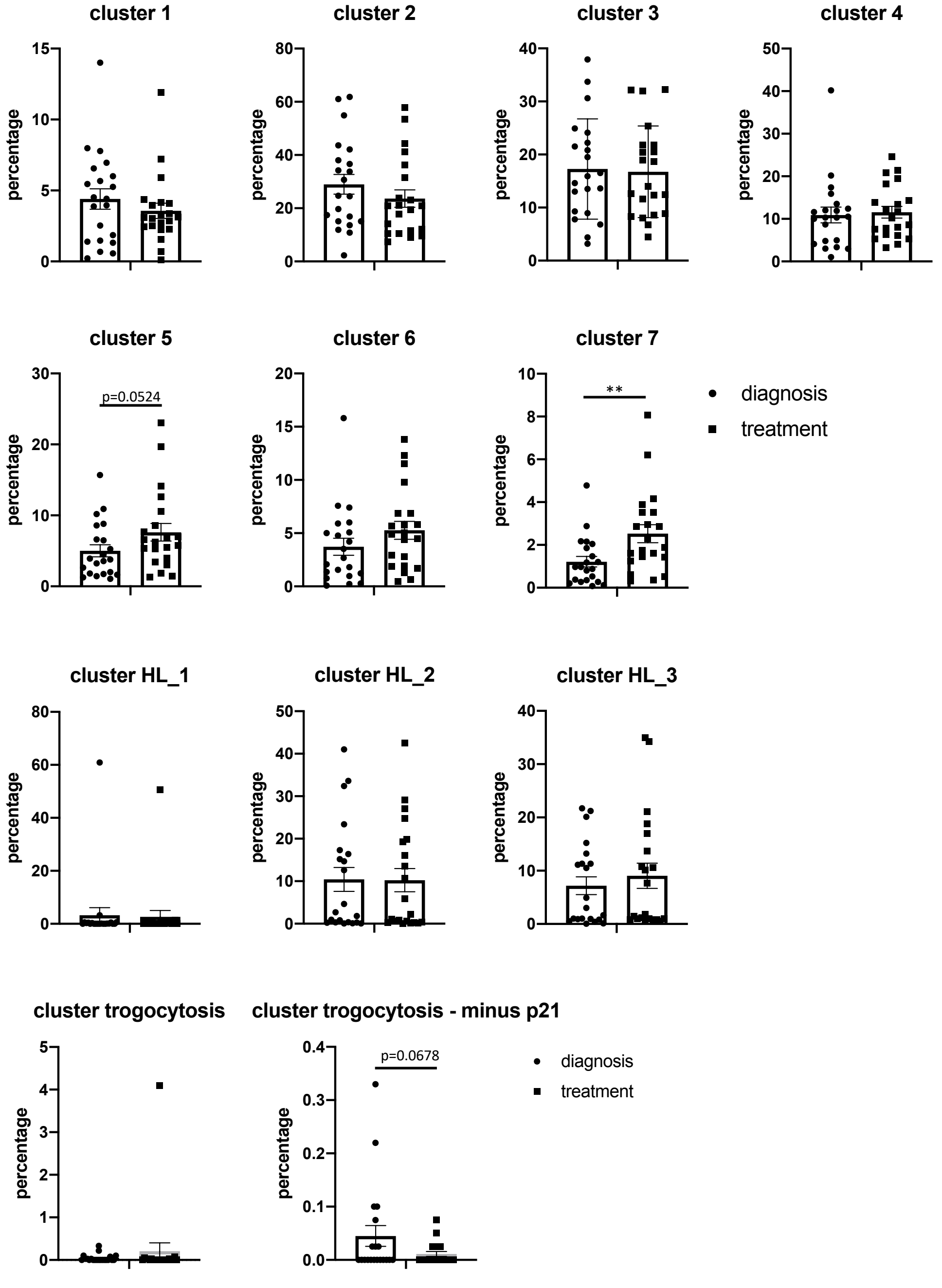

Supplemental Figure 7. Effect of treatment on NK cell clusters in each HL patient. Quantification of the percentage of each cluster before and after treatment for every HL patient.

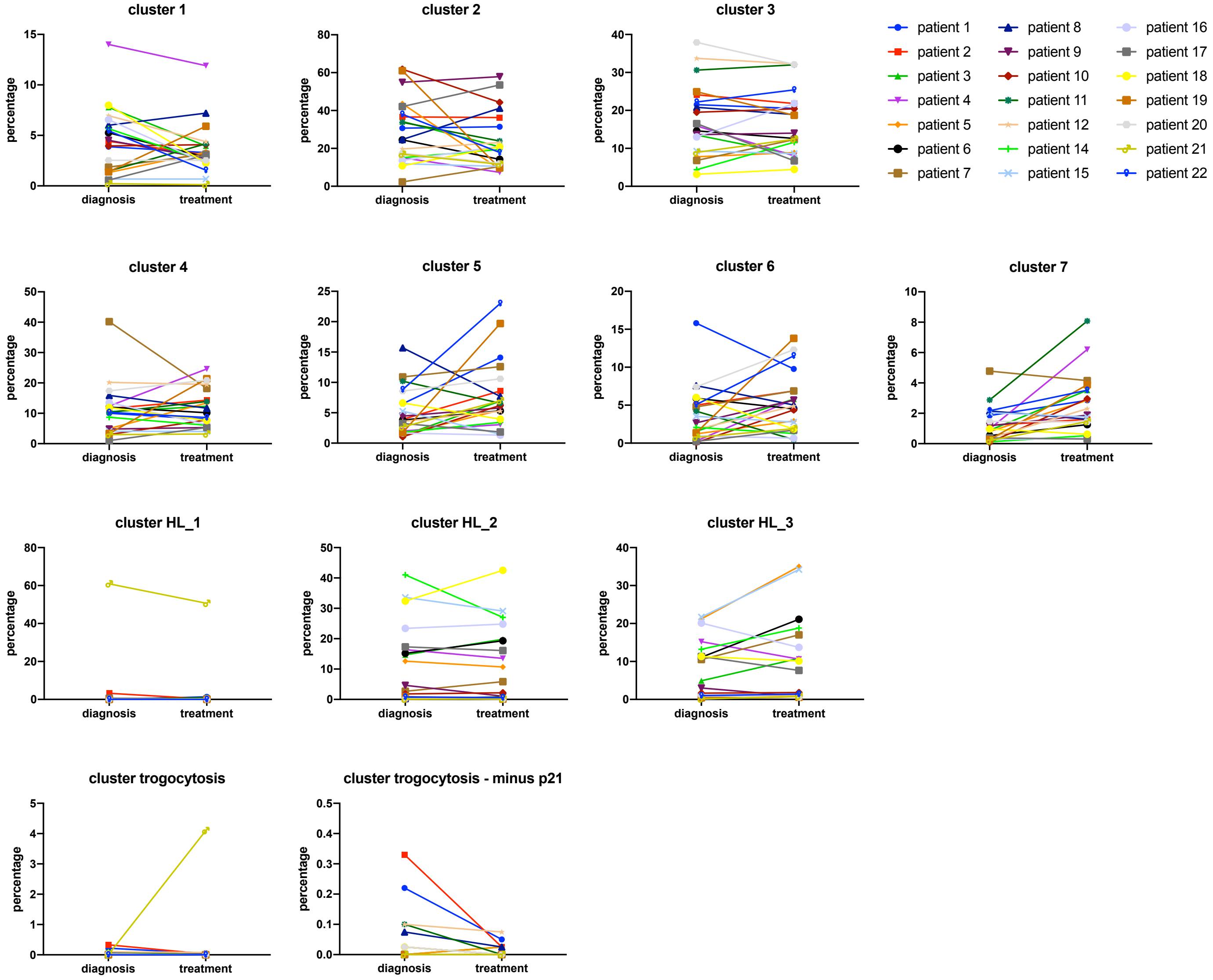

Supplemental Figure 8. Correlation matrix between NK cells and tumor cells. Correlation between the proportion of different NK cell clusters and the total NK and tumo cell numbers by Pearson correlation analysis with correlation coefficient from -1 (negative correlation) to 1 (positive correlation) and confident interval Cl = 95%.

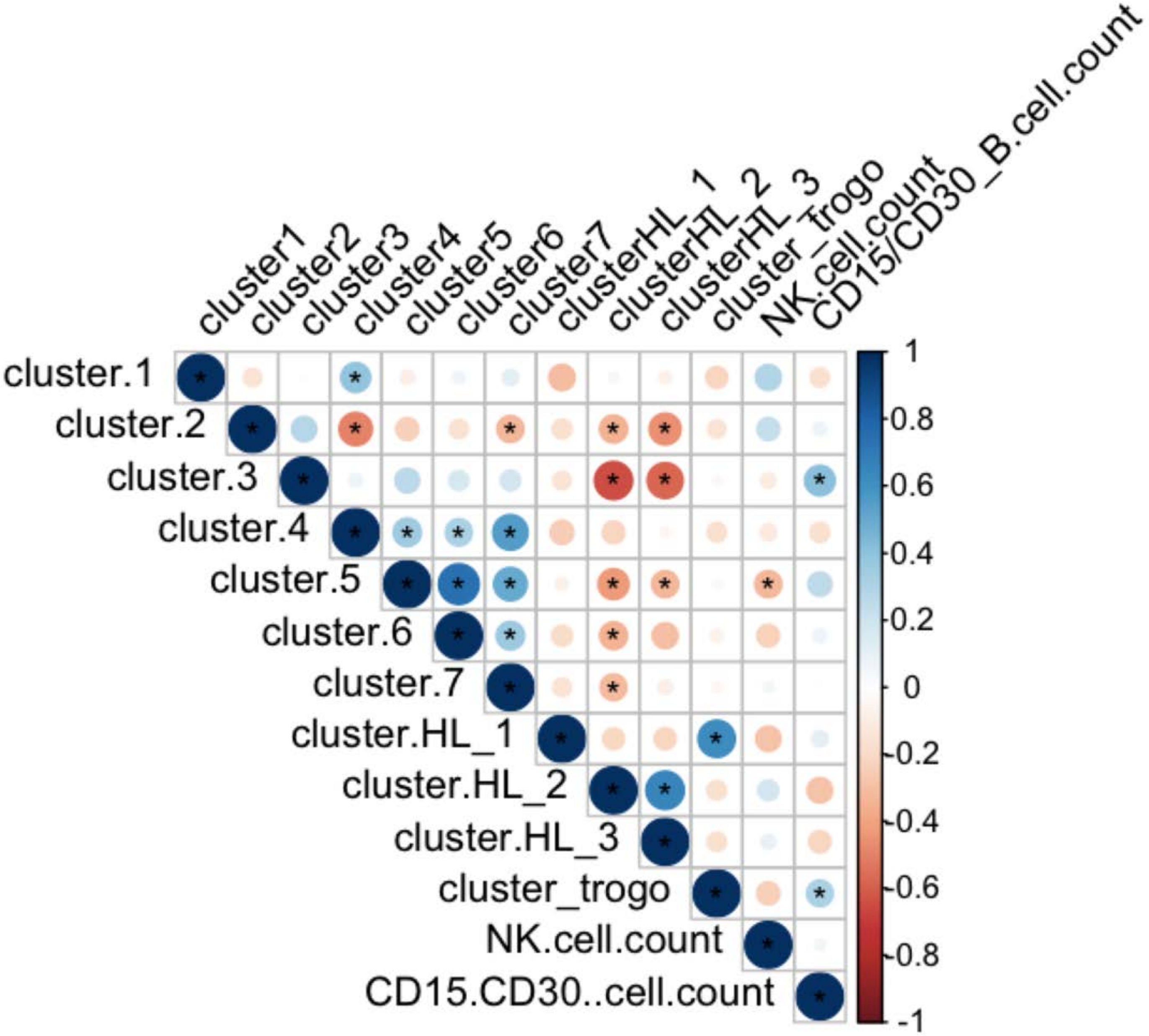

Supplemental Figure 9. NK cell subset analysis stratified by CMV serotypes by manual gating. NK cell phenotypes were analyzed regarding the expression of CD56 and CD16 (A), or CD45RA and CD45R0 (B) between CMV- and CMV+ patients (supplemental table 1). All statistical analysis determined by paired t-test, * p ≤ 0.05, ** p ≤ 0.01 and *** p ≤ 0.001.

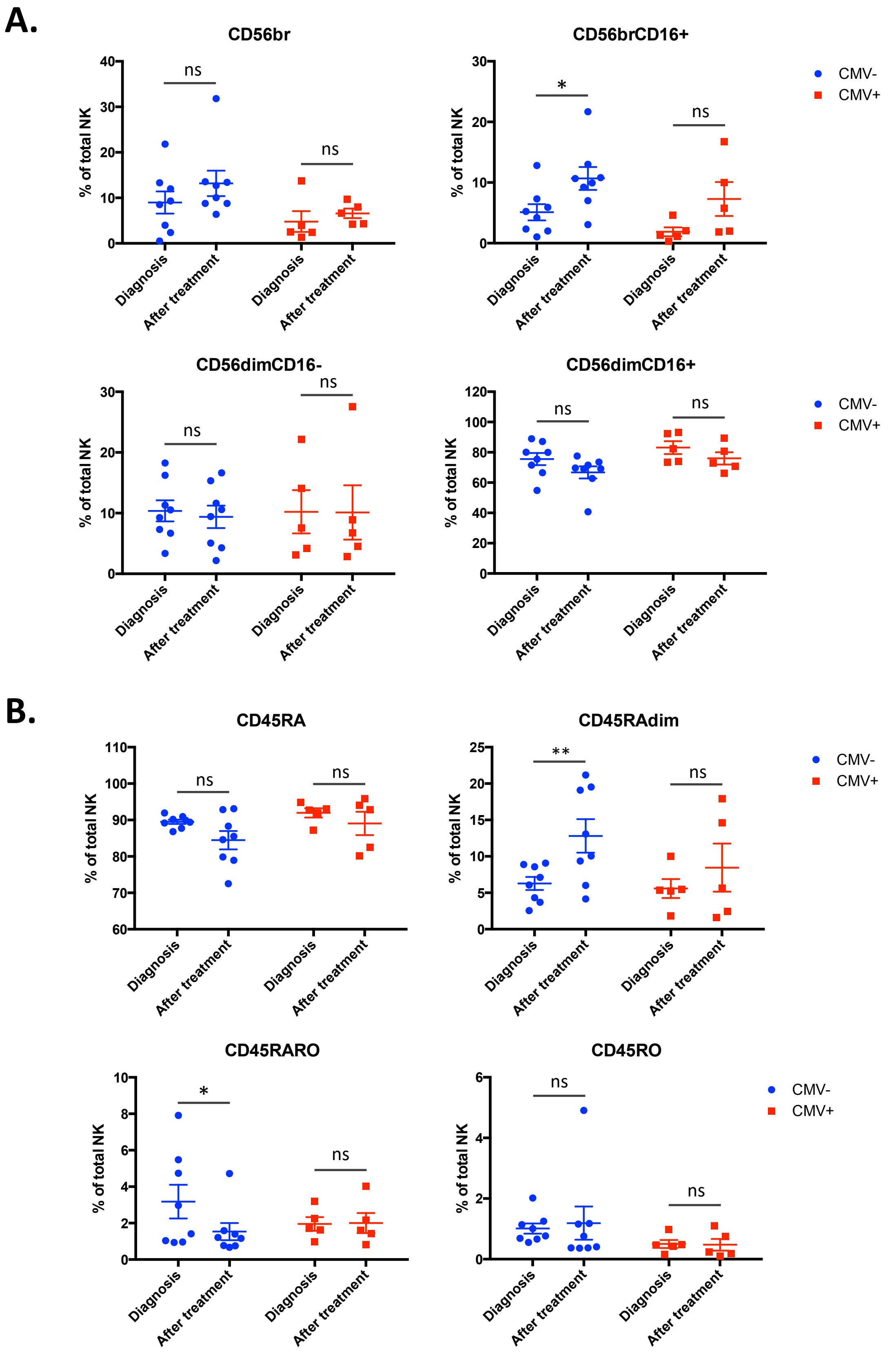

Supplemental Figure 10. NK cell phenotypes from patients stratified by CMV serotypes by manual gating. A) Expression level of PD-1, CD69, NKG2C, CD57, CD62L and CD107a (degranulation ex vivo) on NK cells of CMV- and CMV+ subgroups before and after treatment. B) Trogocytosis on tumor surface markers (i.e, CD19 (negative control), CD15 and CD30) by NK cells before and after treatment between the 2 CMV serotypes. Statistical significance of [diagnosis vs treatment] within CMV groups, and between CMV groups determined by paired t-test and one-way ANOVA (Sidak's correction) respectively , * p ≤ 0.05, ** p ≤ 0.01 and *** p ≤ 0.001.

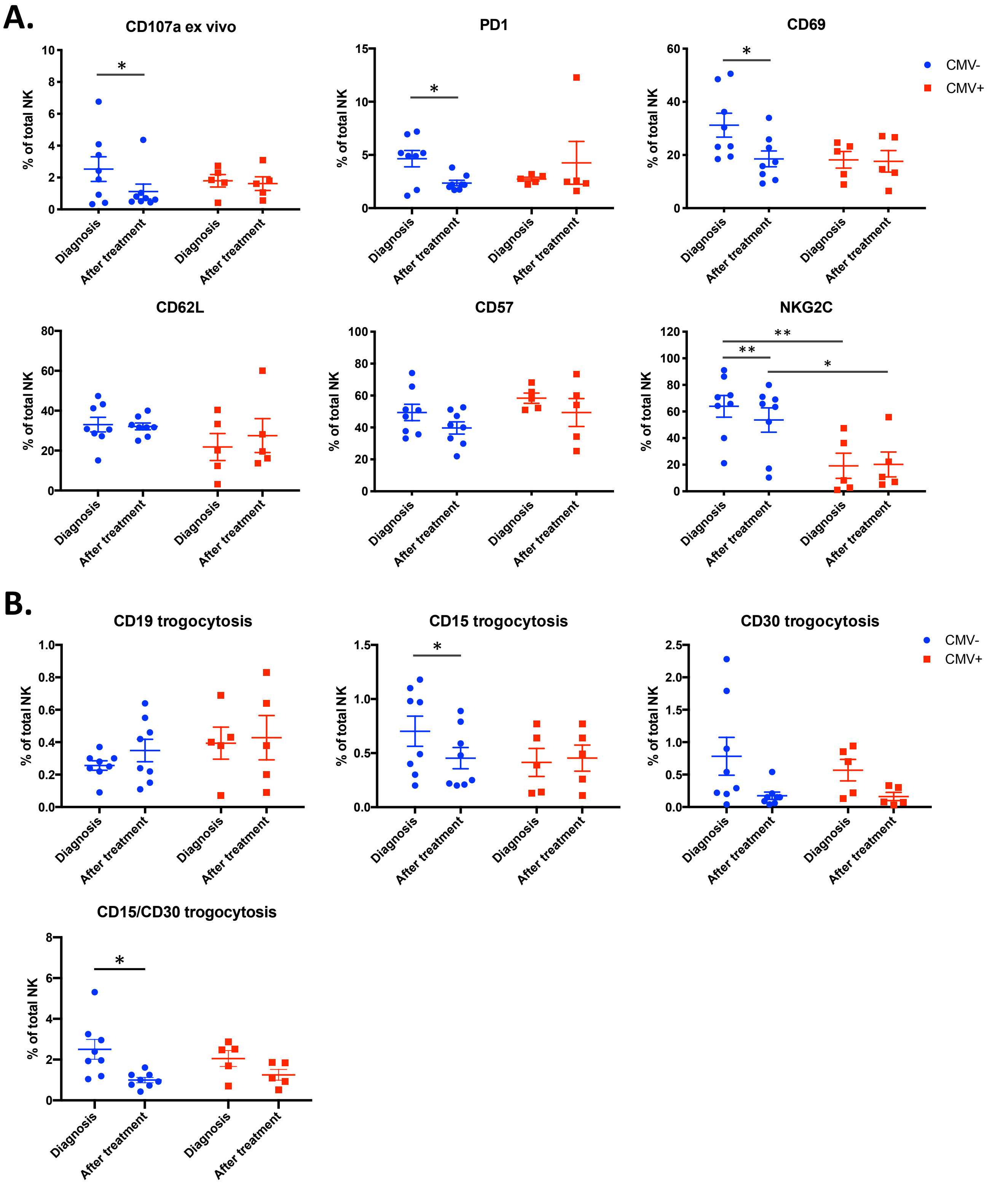

Supplemental Figure 11. Phenotype of NK cell clusters during disease progression. NK cell phenotype was analyzed through 3 timepoints: at diagnosis, after treatment and at relapsed in 2 patients (patient 4 and patient 8). A) Patient 4: UMAP plots of NK cell in each timepoint and quantification of cluster from each timepoint respectively. B) Patient 8 analized as shown in A.

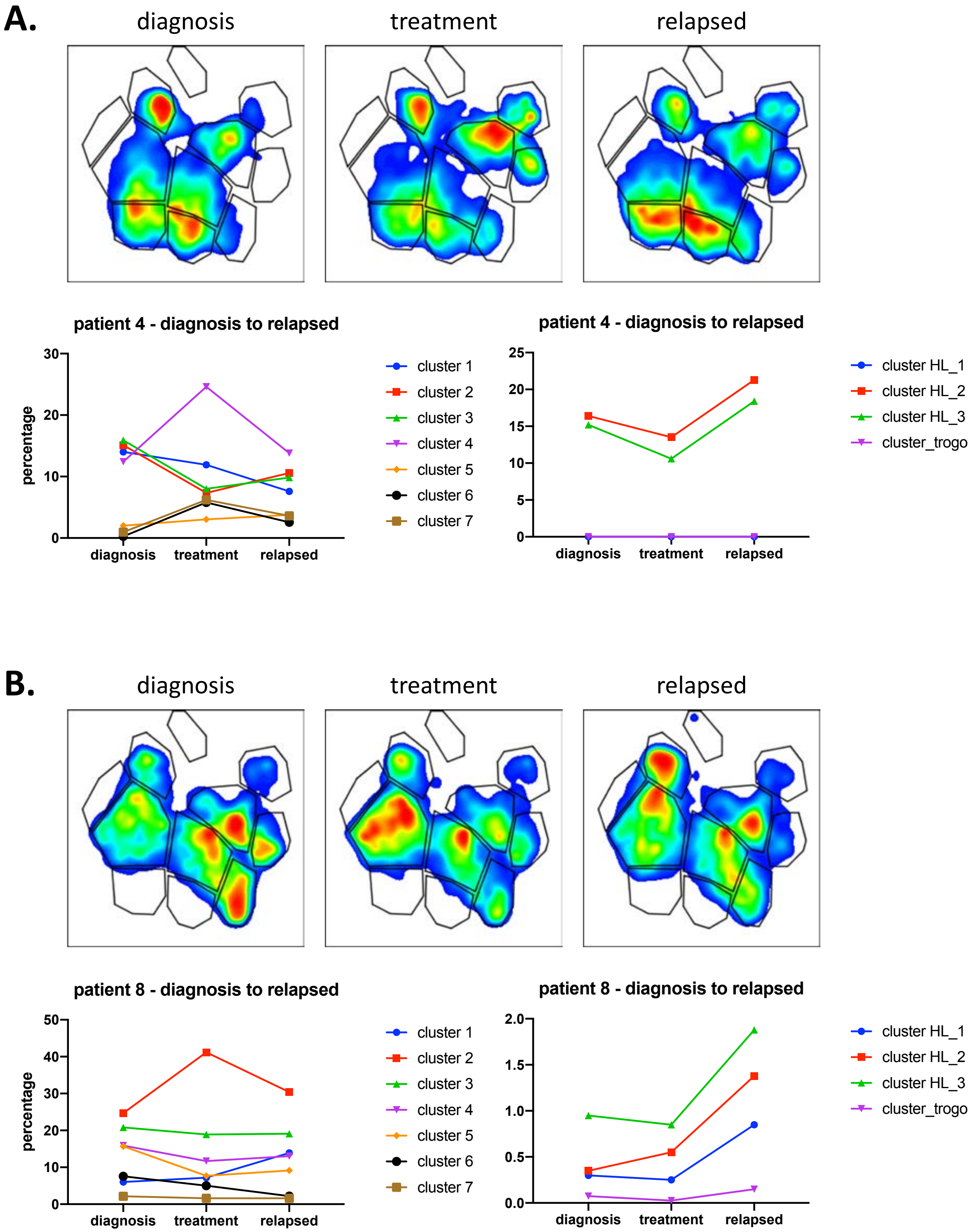

Supplemental Figure 12. Changes in some immune subsets after treatment in an AML cohort of 6 patients. Two samples of each patient (diagnosis and post-induction) with an equal number of cells gated on CD19- compartment was analyzed for the percentage of myeloid blasts (CD33+CD14-), T (CD3+CD56-) and NK (CD3-CD56+) cells. A) Total number of different immune cell populations. B) tSNE plots of each individual patient. CD33 level is shown by color changes. Interestingly, after relapse CD33+ leukemic blasts reappeared in patient 2. The gated regions correspond to T and NK populations.

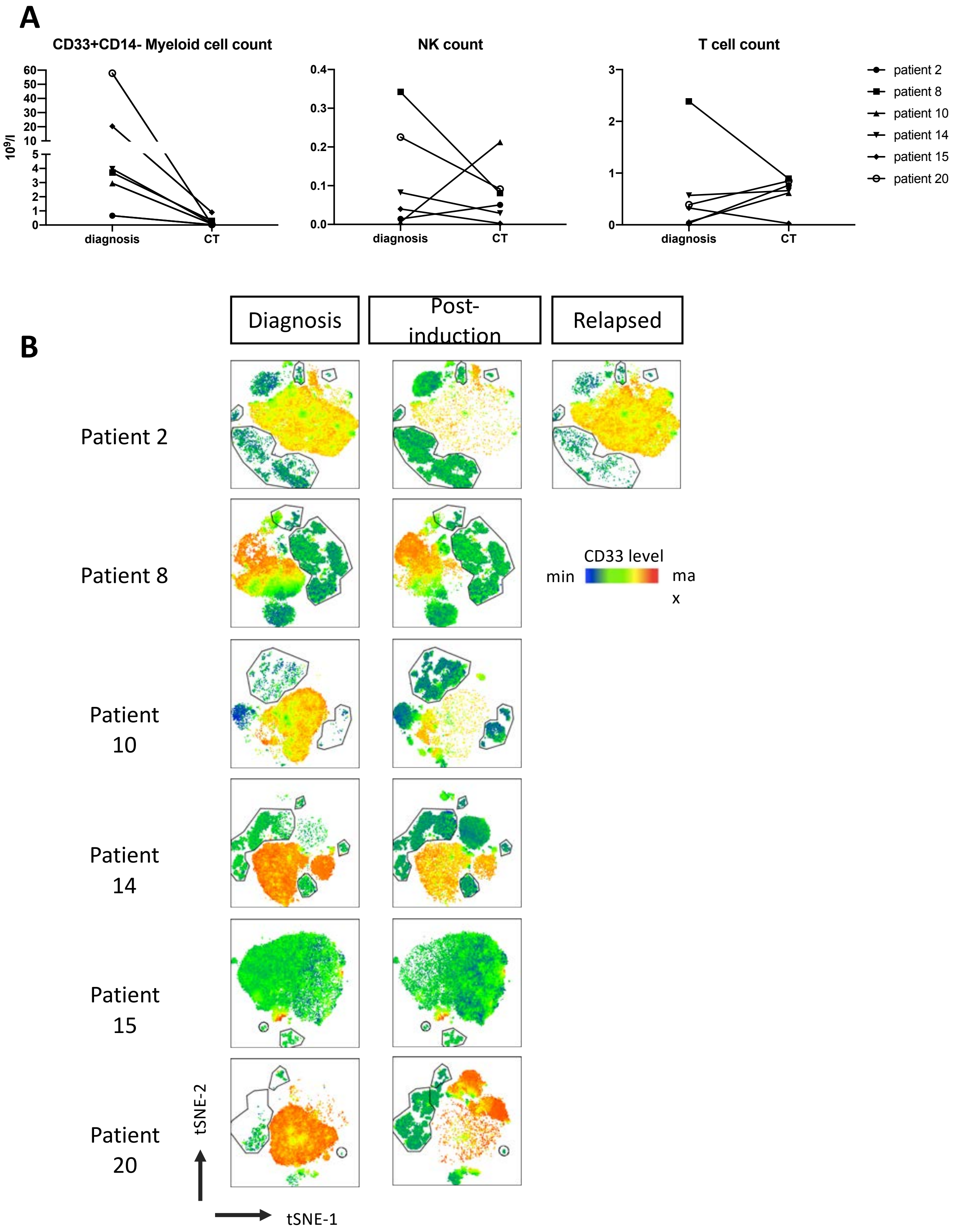

Supplementary Figure 13. Expression level of the 12 surface markers used to generate the UMAP plots on NK cells derived from AML patients. Heatmaps of the expression level of the 12 surface markers that have been used to constitute the UMAP on Figure 6.

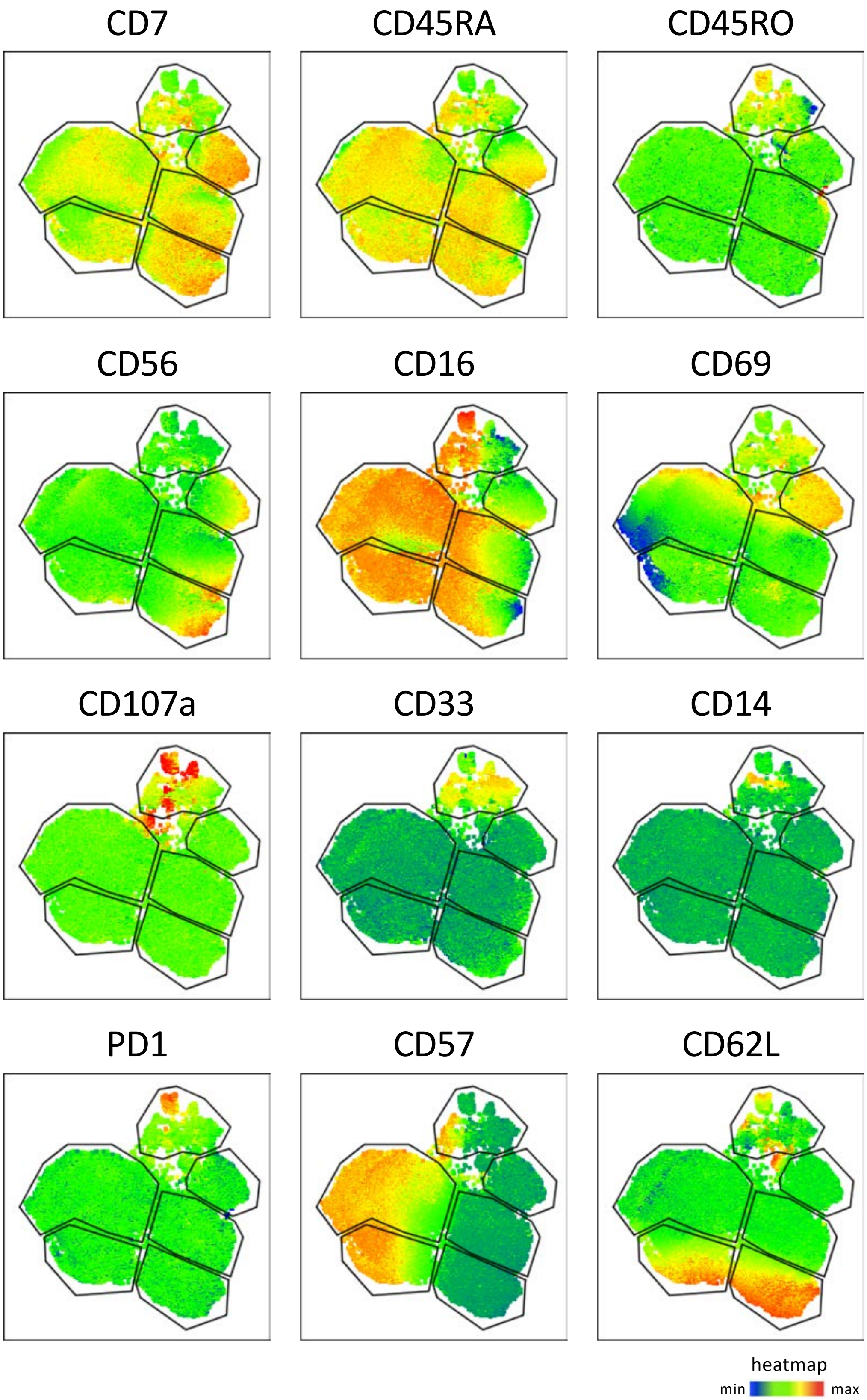

Supplemental Fig. 14. Correlation matrix between different immune subsets and NK cell clusters. Correlation between the percentage of leukemic blasts (CD33+CD14-) and several immune subsets and NK cell clusters for all samples (A) , at diagnosis (B) and after treatment (C) by Pearson correlation analysis with correlation coefficient from -1 (negative correlation) to 1 (positive correlation) and confident interval CI = 95%.

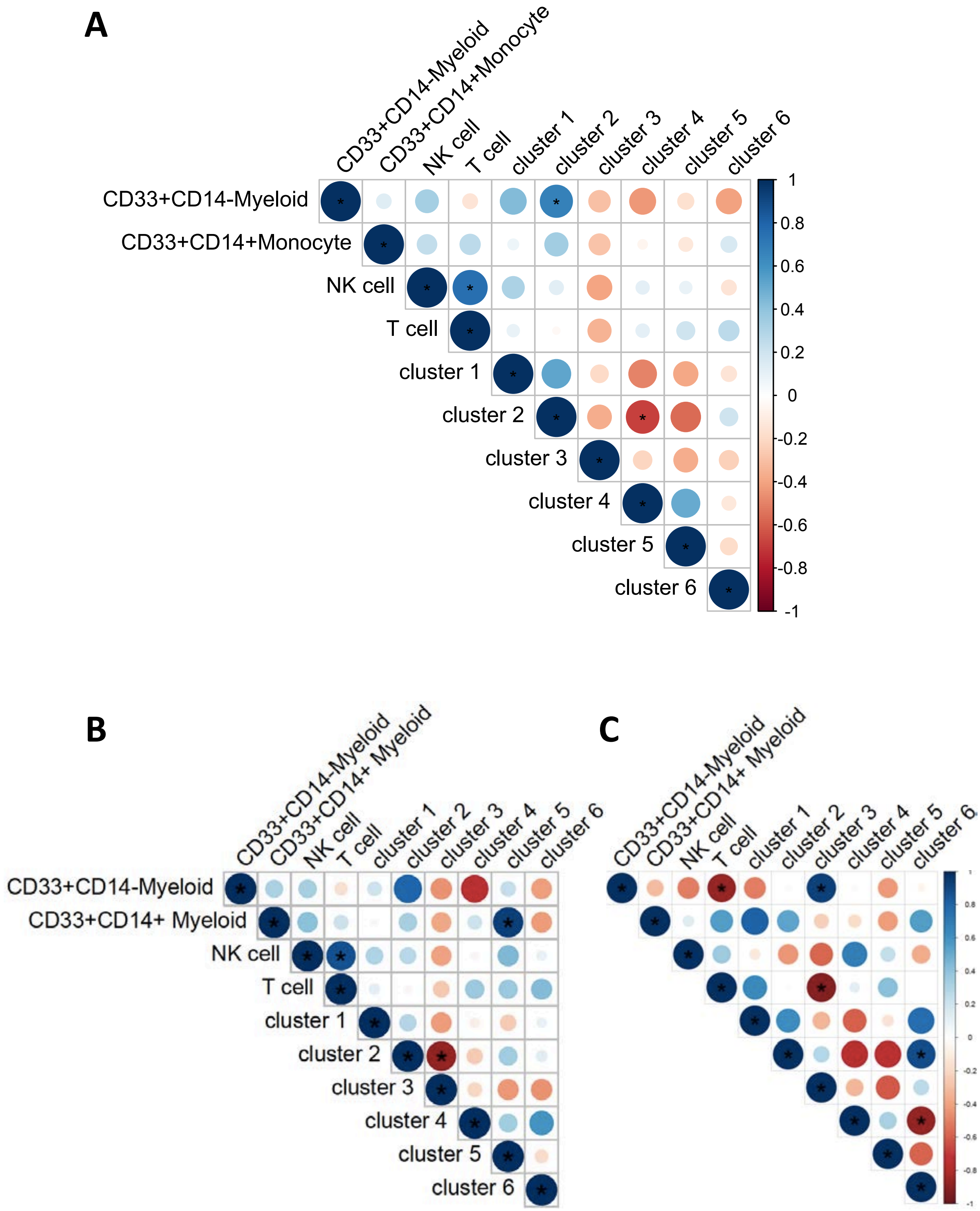

Supplemental Figure 15. NK cell clusters in AML patient 2 after relapse. Transition of NK cell phenotype during disease progression: UMAP plot generated from 6.000 NK from each sample (diagnosis, CT and relapsed) and phenotypically characterization of each cluster. A) original UMAP maps of all samples combined and each timepoints. B) Quantification of clusters identified in (A). C) Ex vivo expression of activation markers (left) and CD45 profile (right) in every clusters.

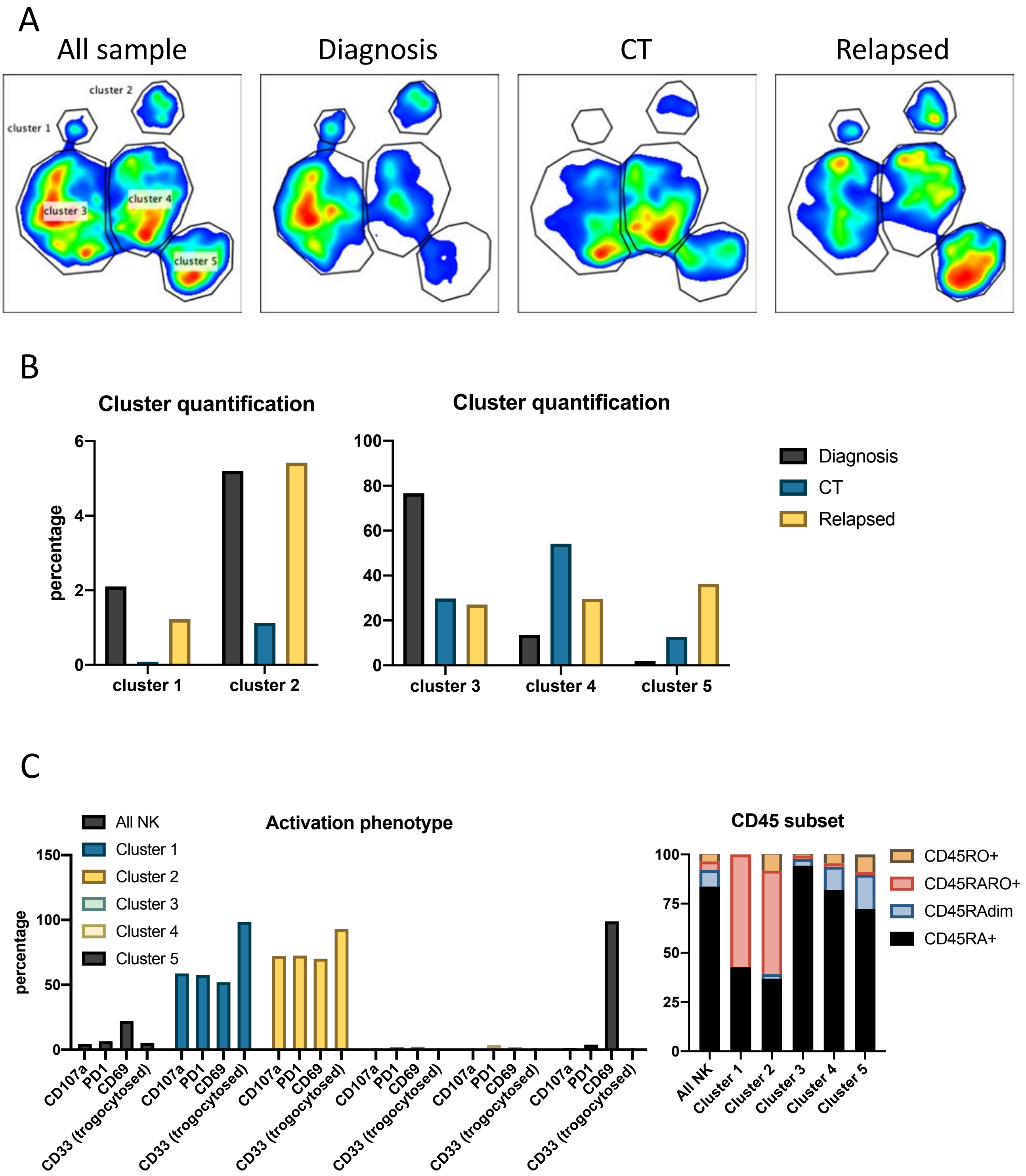

Supplement: Supplementary file 1 [file vaccines-08-00727-s001.pdf]
